# Awareness of and practice toward cancer prevention recommendations: results of the Korean National Cancer Prevention Awareness and Practice Survey in 2021

**DOI:** 10.4178/epih.e2022068

**Published:** 2022-08-26

**Authors:** Jin-Kyoung Oh, Eunjung Park, Byungmi Kim, Yoon-Jung Choi, E Hwa Yun, Min Kyung Lim, Jeong-Soo Im, Eun Young Park

**Affiliations:** 1 National Cancer Control Institute, National Cancer Center, Goyang, Korea; 2 Graduate School of Cancer Science and Policy, National Cancer Center, Goyang, Korea

**Keywords:** Cancer, Primary prevention, Awareness, Practice, Korea, COVID-19

## Abstract

**OBJECTIVES:**

This study reports data regarding the awareness and practice of cancer prevention among Koreans in 2021 and behavioral changes during the coronavirus disease 2019 (COVID-19) pandemic.

**METHODS:**

We collected Cancer Prevention Awareness and Practice Survey data through face-to-face interview surveys using a structured questionnaire completed by 4,000 randomly selected men and women aged between 20 years and 74 years in 17 provinces. We examined the awareness and practice of 10 cancer prevention recommendations and evaluated their associations with potential risk factors through multiple logistic regression analysis adjusted for age, gender, residence, marital status, education, and income.

**RESULTS:**

Eighty percent of participants knew that cancer is preventable, while 45% practiced cancer prevention. Cancer prevention practice tended to be more common among older participants (adjusted odds ratio [aOR], 1.39 per 10-year increment; 95% confidence interval [CI], 1.29 to 1.49) and less common among rural inhabitants (aOR, 0.66; 95% CI, 0.51 to 0.86) than among urban residents and among single people (aOR, 0.55; 95% CI, 0.45 to 0.66) than among married people. Practices were the highest for avoiding burned or charred foods (87.6%) and lowest for vaccination against human papillomavirus (14.5%). Refusal to follow recommendations was most common for avoiding alcohol consumption (7.9%). The most difficult recommendations to follow were (1) regular exercise (57.7%); (2) maintaining a healthy body weight (46.1%); and (3) avoiding alcohol (40.1%). The most significant COVID-19-related changes were less exercise (32.5%) and increased body weight (25.6%).

**CONCLUSIONS:**

The awareness of cancer prevention was high, but the practice was low. Recommendations targeting awareness and practice need to be further promoted.

## INTRODUCTION

Cancer is a leading cause of death worldwide, accounting for approximately 10 million deaths in 2020 [1]. In the Korean, cancer accounts for 27.5% of all deaths, followed by heart disease, cerebrovascular disease, and pneumonia. In 2019, the age-standardized cancer incidence and mortality rates were 275.4 and 72.2 per 100,000, respectively [2]. Tobacco use, alcohol consumption, unhealthy diets, physical inactivity, and chronic infections are well-known risk factors for cancer. Approximately 30-40% of cancers are preventable by avoiding risk factors and implementing preventive strategies [3]. Successful cancer prevention requires a combination of individual (avoiding or reducing harmful exposures) and group actions (eliminating or reducing exposures by population-level measures) [4].

Individuals need to understand the relevant evidence to reduce their cancer risk. Several health agencies provide cancer prevention guidelines to encourage people to avoid or reduce harmful exposures, such as the European Code against Cancer [4], American Cancer Society Guidelines for Cancer Prevention [5], and the Cancer Prevention Recommendations by the World Cancer Research Fund in collaboration with the American Institute for Cancer Research [6]. The Korean National Codes against Cancer are a set of recommendations providing advice on cancer prevention to the public. The first edition was published in 2006 by the Korea Ministry of Health and Welfare in collaboration with the National Cancer Center and various academic societies [7]. It lists 10 lifestyle recommendations to improve general health and prevent cancer: do not smoke and avoid secondhand smoke, maintain a healthy diet, reduce salt intake, avoid burnt or charred foods, limit alcohol consumption, engage in physical activity, maintain a healthy body weight, receive hepatitis B virus (HBV) vaccine, follow safety measures to avoid carcinogens in the workplace, and receive regular cancer screenings. Its second edition, published in 2016 [8], also recommends avoiding alcohol consumption and receiving the human papillomavirus (HPV) vaccination (Supplementary Material 1).

To monitor Korean citizens’ awareness levels and cancer prevention practices, the Cancer Prevention Awareness Survey (CPAS) has been conducted periodically since 2007. In this study, we aimed to report the 2021 results for 10 cancer prevention recommendations and their 15-year trends and examine changes in cancer prevention behaviors due to the coronavirus disease 2019 (COVID-19) pandemic.

## MATERIALS AND METHODS

### Data source

We used CPAS data collected between 2007 and 2021. The National Cancer has conducted the CPAS annually (2007-2010) and biannually (2012-2021). It is a nationwide, population-based cross-sectional survey that employs a proportional quota sampling design based on the resident population according to geographic area, age, and gender. Data were collected through face-to-face interviews conducted by a professional research agency. The sample size was 1,000-2,000 in each survey between 2007 and 2018. The latest survey, conducted between November 25, 2021 and February 7, 2022, included 4,000 randomly selected men and women aged between 20 years and 74 years in 17 provinces. The response rate for the 2021 survey was 17.1%; among the 23,450 target population, 40.2% refused to participate, 39.3% failed to be contacted, 2.4% were not eligible, and 1.0% withdrew participation during the interview. The response rates of previous surveys were unavailable but could be higher considering that face-to-face interviews were restricted due to social distancing during the COVID-19 pandemic.

### Assessing awareness and practice of cancer prevention

Using a structured questionnaire, we asked participants about their awareness and practice of cancer prevention in general and about each recommendation: (1) no smoking and avoiding secondhand smoke, (2) consuming a sufficient amount of fruits and vegetables and a balanced diet, (3) reducing salt intake and avoiding burnt or charred foods, (4) limiting alcohol consumption, (5) engaging in regular physical activity (i.e., at least 30 minutes, 5 days a week), (6) maintaining a healthy body weight, (7) immunization against HBV and HPV, (8) undergoing regular cancer screening, and (9) engaging in safe gender, which includes not having gender, having a single sexual partner, and using condoms. We did not ask about the recommendation regarding workplace safety awareness or practices due to a historically small number of survey participants working with cancer-causing agents.

We developed the questionnaire based on the Precaution Adoption Process Model (PAPM). The PAPM is a theory for understanding how people come to take action to prevent illnesses, injuries, or other types of harm. Its underlying assumption is that people must pass through a series of 6 qualitatively distinct stages on the path from ignorance to action. The PAPM posits that preventive health behavior can include 6 nominal intention stages: (1) *unaware* of the health behavior; (2) *unengaged* in the decision; (3) *undecided*; (4) *decided not to act*; (5) *decided to act*; and (6) *acting* [9]. We categorized the PAPM stages into 3 groups: (1) pre-adoption (stages 1-3); (2) refusal (stage 4); and (3) adoption (stages 5 and 6).

We assessed the awareness of overall cancer prevention with the question “Do you think cancer is preventable?” and the practice of cancer prevention with “Have you ever tried to prevent cancer?” For cancer prevention recommendation-specific awareness and practice, we used the questions: “Do you think [risk/preventive factor] causes/prevents cancer?” and “Do you practice [risk/preventive behavior]?” For example, a question on the awareness of physical activity was, “Do you think physical activity (i.e., at least 30 minutes a day, 5 days a week) prevents cancer?” A question on the practice of physical activity was, “Are you physically active in your daily life?” PAPM stages 2-6 were assessed among respondents who were aware of each risk/preventive factor, while “practice” was assessed among all participants.

To investigate changes in health behaviors due to the COVID-19 pandemic, we calculated the proportion of participants for whom health behaviors changed for 7 recommendations: smoking, alcohol consumption, diet, physical activity, body weight, vaccination, and cancer screening. For example, we asked, “In comparison with the years before the COVID-19 pandemic, has your smoking behavior changed?” and possible responses were (1) did not change, (2) changed but not because of COVID-19, (3) quit smoking because of COVID-19, and (4) started smoking due to COVID-19. The structure of questions and options for alcohol consumption, diet, physical activity, body weight, and cancer screening were the same. For immunization programs, we asked, “Compared to the years before the COVID-19 pandemic, has your attitude toward vaccinations changed, not only for COVID-19 vaccinations but for other vaccination programs in general?” and the options were (1) unchanged, (2) had trusted vaccinations before, and trusted them more firmly during the pandemic, (3) had distrusted vaccinations before but came to trust them during the pandemic, (4) had distrusted vaccinations before, and distrusted them even more during the pandemic, and (5) had trusted vaccinations before but came to distrust them during the pandemic.

### Statistical analysis

We considered the following as potentially related factors: age (ranges: 20-29, 30-39, 40-49, 50-59, and 60-74 years), gender, residential area (metropolis, small and medium cities, countryside), region (Seoul, Busan, Daegu, Incheon, Gwangju, Daejeon, Ulsan, Sejong, Gyeonggi, Gangwon, Chungbuk, Chungnam, Jeonbuk, Jeonnam, Gyeongbuk, Gyeongnam, and Jeju), marital status (married, single, and widowed/separated/divorced), educational level (middle school or less, high school, and college or more), and monthly income (less than 3 million Korean won [KRW]; 3 million KRW to less than 6 million KRW; and 6 million KRW or more). As a continuous variable, age was entered into the models in 10-year increments.

To evaluate the associations of potential risk factors with awareness and practice of cancer prevention, we applied multiple logistic regression models to obtain odds ratios (ORs) and 95% confidence intervals (CIs) mutually adjusted for age, gender, residential area, marital status, education level, and monthly income.

The proportions of participants who were aware of and implemented cancer prevention recommendations were expressed as crude and age-standardized rates (ASRs). We calculated ASRs (a weighted average of age-specific rates, where the weights represent the proportions of people in the corresponding age groups of a standard population) to compare proportions across time with different population age structures. Furthermore, we standardized them using the 2015 mid-year population data from Statistics Korea. The observed trends in awareness and practice were estimated using a joinpoint regression model with a maximum of 3 joinpoints [10]. To identify significant changes in ASRs, we summarized the rates of change as a constant amount over time using a linear model for the ASRs. A logarithmic transformation of proportions of awareness and practice was not performed.

All statistical analyses were conducted using SAS version 9.4 (SAS Institute Inc., Cary, NC, USA), R version 4.1.3 (R Core Team, Vienna, Austria), and SEER*Stat, Joinpoint 4.7.0 (National Cancer Institute, Bethesda, MD, USA). Statistical significance was set at p-value < 0.05.

### Ethics statement

This study was approved by the Institutional Review Board of the National Cancer Center, Korea (NCCNCS-07-102, NCC2016-0153, NCC2022-0012).

## RESULTS

### Awareness and practice of cancer prevention in 2021

**Figure 1.**
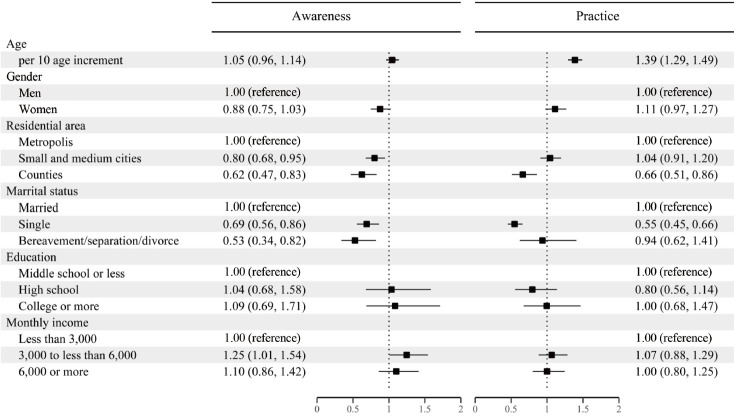
Adjusted odds ratios (aORs) and 95% confidence intervals (CIs) of related factors of awareness and practice of cancer prevention. aORs were mutually adjusted for age, gender, residential area, marital status, education level, and monthly income. Values are presented as aOR (95% CI).

Table 1 shows the characteristics of the study participants in 2021 and the proportions of awareness and practice of cancer prevention. The percentages of people who answered “yes” to the questions “Do you think cancer is preventable?” and “Have you ever tried to prevent cancer?” were 80.3% and 45.0%, respectively. Lower awareness was found in small cities (aOR, 0.80; 95% CI, 0.65 to 0.95) and counties (aOR, 0.62; 95% CI, 0.47 to 0.83) than in large cities, and among single people (aOR, 0.69; 95% CI, 0.56 to 0.86) and those with another marital status (i.e., widowed/separated/divorced) (aOR, 0.53; 95% CI, 0.34 to 0.82) than among married people; however, higher awareness was found in the middle-income group (i.e., 3-6 million KRW monthly income) (aOR, 1.25; 95% CI, 1.01 to 1.54) than in the lower-income group (i.e., < 3 million KRW). Prevention practice was more likely among older participants (aOR, 1.39 per 10 years increment; 95% CI, 1.29 to 1.49) and lower in counties (aOR, 0.66; 95% CI, 0.51 to 0.86) than in large cities and among single people (aOR, 0.55; 95% CI, 0.45 to 0.66) than among married people (Figure 1). We also found regional variations in awareness and prevention levels (Supplementary Material 2). The regional levels of awareness and practice were similar to those observed within Seoul except for Daegu (age- and gender-adjusted OR, 2.0; 95% CI, 1.5 to 2.8 for practice), Gyeonggi (aOR, 1.3; 95% CI, 1.1 to 1.6 for practice), and Gyeongnam (aOR, 1.7; 95% CI, 1.1 to 2.6 for awareness; aOR, 1.6; 95% CI, 1.2 to 2.1 for practice), which had higher levels, and Ulsan (aOR, 0.6; 95% CI, 0.4 to 0.9 for practice), Jeonbuk (aOR, 0.4; 95% CI, 0.3 to 0.6 for awareness), and Gyeongbuk (aOR, 0.7; 95% CI, 0.4 to 0.96 for awareness) with lower levels (Supplementary Material 3).

Table 2 shows information on cancer prevention recommendation-specific awareness, practice, and distribution of PAPM stages. The level of awareness was the highest for avoiding burned or charred foods (95.8%), followed by cancer screening (95.6%), not smoking (95.5%), having a balanced diet (93.9%), and eating fruits and vegetables (92.8%). The level of practice was the highest for avoiding burned or charred foods (87.6%) and the lowest for vaccination against HPV (14.5%). Refusal to follow recommendations (deciding not to act) was most common for avoiding alcohol consumption (7.9%).

**Figure 2.**
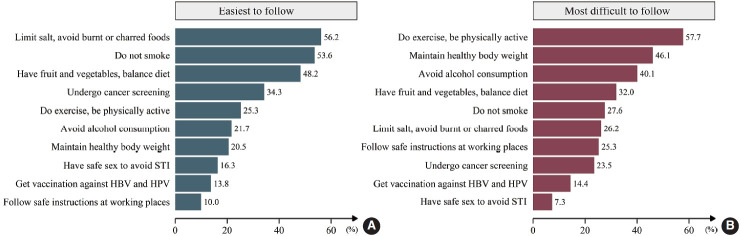
Rates of recommendations selected as easiest (A) and most difficult (B) to follow. HBV, hepatitis B virus; HPV, human papillomavirus; STI, sexually transmitted infection.

The most commonly followed recommendations were (1) limit salt intake and avoid burnt or charred food (56.2%), (2) do not smoke (53.6%), and (3) consume a balanced diet with sufficient fruits and vegetables (48.2%). The most difficult recommendations to follow were (1) exercise regularly (57.7%), (2) maintain a healthy body weight (46.1%), and (3) avoid drinking alcohol (40.1%) (Figure 2).

### Changes in awareness and practice over time

**Figure 3.**
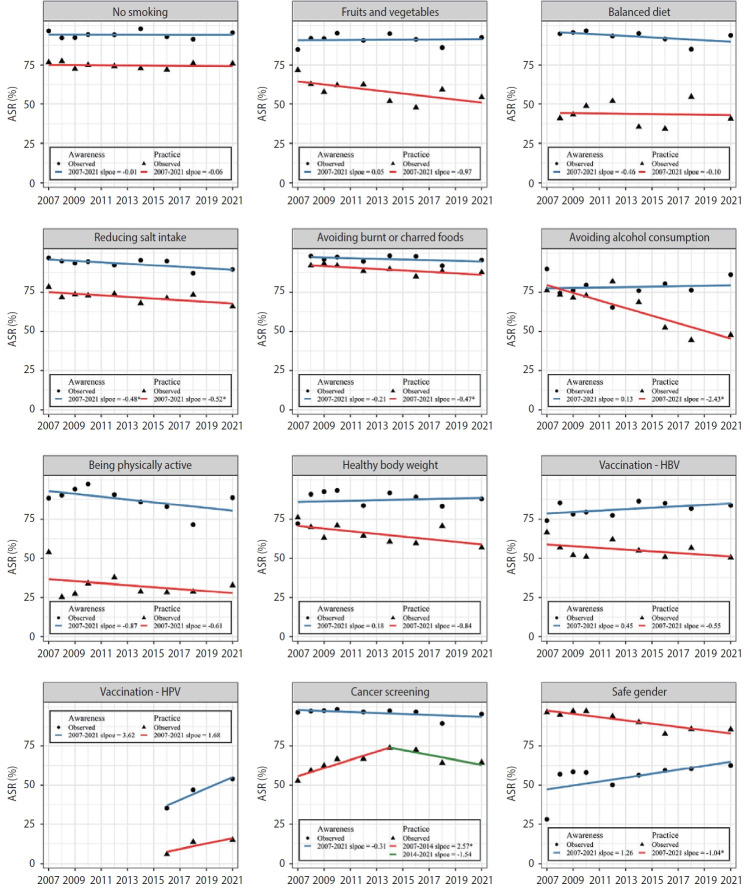
Awareness and practice of each cancer prevention recommendation, 2007-2021. ASR, age-standardized rates; HBV, hepatitis B virus; HPV, human papillomavirus. ^*^p<0.05.

The proportion of participants with cancer prevention awareness decreased from 84.3% in 2007 to 80.3% in 2021, while prevention practice increased from 41.0% to 45.0%. However, these changes were statistically insignificant (Supplementary Material 4). The awareness of HPV vaccination significantly increased from 36.9% in 2016 to 50.2% in 2021 after the HPV vaccine was introduced into the National Immunization Program (NIP) and added to cancer prevention recommendations in 2016. The practice of limiting/avoiding alcohol consumption decreased (annual percent change, -2.43%; p< 0.05) even before 2016, when the recommendation was revised from limiting alcohol consumption to 1-2 drinks to avoiding alcohol consumption entirely. The practice of reducing salt intake, avoiding burnt or charred foods, and having safe gender slightly decreased. Cancer screenings increased until 2014 (annual percent change, 2.57%; p< 0.05) and were stagnant thereafter (Figure 3).

### Effect of the COVID-19 pandemic on health behaviors

**Figure 4.**
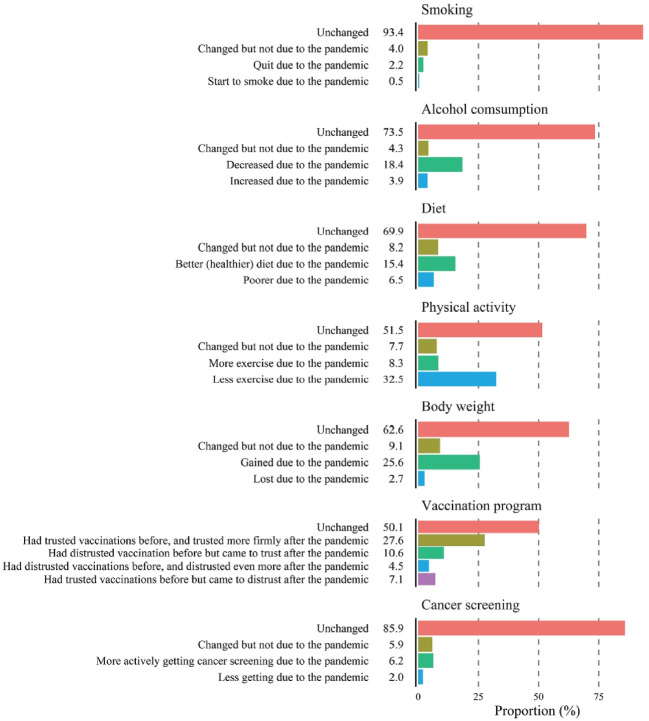
Changes in health behaviors during the coronavirus disease 2019 pandemic.

Figure 4 shows the effect of the COVID-19 pandemic on health behaviors. The most significant changes due to COVID-19 were less exercise (32.5%) and increased body weight (25.6%). Only a small proportion of study participants (2.2%) quit or started smoking (0.5%) due to COVID-19. While 18.4% of participants consumed less alcohol, 3.9% drank more due to COVID-19. Moreover, 15.4% of respondents were more likely to eat a balanced diet, while 6.5% ate worse during the COVID-19 pandemic. A considerable proportion of participants (27.6%) trusted vaccinations before and trusted them more firmly after the pandemic, whereas 10.6% distrusted vaccinations before but began to trust vaccinations after the pandemic. Approximately 4.5% of participants distrusted vaccinations before and came to distrust them even more after the pandemic. The remaining 7.1% of participants trusted vaccinations but came to distrust them due to COVID-19. For cancer screening, 6.2% of participants became more engaged due to COVID-19, while 2.0% of participants became less participatory in cancer screening.

## DISCUSSION

In other countries, adherence to cancer prevention recommendations has been found to substantially reduce the risk of cancer [11-13]. In our study, participants were highly aware (80.3%) of cancer prevention, but only half (45.0%) of them practiced it daily. The level of awareness and practice regarding cancer prevention has not changed significantly over the last 15 years, even though various campaigns and social events to raise awareness have been conducted. In Korea, after publishing the national recommendations on cancer prevention in 2006, cancer prevention campaigns were initiated by the government, community, and academic groups. For example, public events such as the annual Cancer Prevention Day (March 21, enacted in 2006) attempt to raise cancer prevention awareness [14]. Celebrities appointed as cancer prevention ambassadors also participate in media campaigns [15]. Detailed information and guidelines on cancer prevention are provided by the National Cancer Information Center (http://www.cancer.go.kr) and various media channels. Despite these efforts, cancer prevention awareness and practice have not improved overall. Health promotion and cancer prevention programs should be expanded, especially those targeting young people and people living in small cities and counties who are less likely to engage in cancer prevention behaviors.

Among the 10 recommendations, “do not smoke and avoid secondhand smoke,” “balanced diet,” and “reduced salt and burnt or charred food intake” were the easiest to follow and showed the highest proportions of awareness and practice. Comprehensive tobacco control measures have been implemented in Korea by the Health Promotion Act, including anti-tobacco campaigns, pictorial warnings on cigarette packaging, regulations to protect people from secondhand smoke, and raising tobacco tax [16]. Those policies and programs contributed to tobacco denormalization, and as a result, the smoking rate declined from 28.8% in 2005 to 20.6% in 2020 according to the Korea National Health and Nutrition Examination Survey (KNHANES) [17,18]. In our study, the proportion of smokers fluctuated between 22.6% in 2008 and 27.9% in 2016 without significant increasing or decreasing trends. To promote healthy diets, the Ministry of Food and Drug Safety of Korea established comprehensive measures for sodium reduction in 2012 and implemented programs to reduce sodium intake, including public awareness campaigns and establishing low-sodium food consumption environments in restaurants and meal services. The daily sodium intake per person decreased by approximately 30%, from 4,789 mg in 2010 to 3,189 mg in 2020 [18], but remains higher than the World Health Organization’s recommendation of 2,000 mg [19].

In contrast, “exercise regularly and be physically active,” “maintain a healthy body weight,” and “avoid alcohol consumption” were selected as recommendations that were challenging to follow. Promoting those healthy habits in daily life at the individual level and establishing healthy environments at the community level are needed. Alcoholic beverages are widely consumed in Korea; according to KNHANES, the prevalence of alcohol drinking (i.e., the percentage of adults who have had alcoholic drinks 1 or more times a month during the past year) gradually increased from 54.6% in 2005 to 58.9% in 2020 [17,18]. Even though several studies have suggested the classic J-shaped curve regarding the protective effect of light alcohol consumption for ischemic heart disease and all-cause mortality [20,21], it is well established that there is no safe limit of alcohol consumption for causing cancer [22,23]. Based on the scientific evidence [5,24], Korean cancer prevention recommendations have been revised to “not drinking alcohol” from “limit alcohol consumption to 1-2 drinks per day.” After revising the alcohol recommendation in 2016, the survey question was changed from “Do you abstain from alcohol or consume only 1-2 drinks per day?” to “Do you abstain from alcohol?” The proportion of respondents following this recommendation decreased from 76.2% in 2007 to 47.7% in 2021, while the awareness of alcohol as a cancer risk factor remained stable. The change in the definition of alcohol-related practice and the actual increase in alcohol drinking prevalence might have contributed to the abrupt decline in the proportion of people following the recommendation. The high proportion of people practicing alcohol-related recommendations in previous surveys suggests that there is a large number of light alcohol drinkers who are potential targets for intervention. The prevalence of high-risk alcohol consumption (i.e., the percentage of adults who drink ≥ 7 [men] or ≥ 5 [women] drinks more than 2 times a week) was reported to be 14.1% in 2020 [18]. Based on these prevalence rates, we can estimate that approximately 45% of adults in the general population drink alcohol at light-moderate levels (< 7 [men] or < 5 [women] drinks, less than twice a week). In our study, 39% of our study participants were in the preadoption stage (unaware, unengaged, or undecided) and 8% refused (i.e., decided not to act) to follow the recommendation for avoiding alcohol consumption; common reasons given were “enjoying drinking” (56%) and “difficult to refuse drinking at a party or social gathering” (24%).

Study participants were highly (89%) aware of the benefit of physical activity for cancer prevention, although only 32.5% regularly exercised for at least 30 minutes, 5 days a week. This was slightly lower than the proportion of people who walked for at least 30 minutes, 5 days a week (39.2% in 2020) as reported by the KNHANES. According to the KNHANES, participation in aerobic physical activities, which is defined as the percentage of adults who perform 150 minutes of moderate-intensity physical activities or 75 minutes of vigorous-intensity physical activities or an equivalent combination of moderate-intensity and vigorous-intensity physical activities per week, has declined among Koreans, from 72.5% in 2007 to 47.6% in 2018 in both genders [17]. In our study, the main reasons for not performing physical activity were “having no time” (52%) and “not enjoying it” (33%) (data not shown).

Similarly, people were aware of the benefits of maintaining a healthy body weight but perceived it as a challenge because of “uncontrolled diet due to appetite and frequent dining out” (43%) and “no time to exercise” (22%) (data not shown). The prevalence of obesity in Korea (body mass index ≥ 25 kg/m^2^) increased from 31% in 2010 to 38% in 2020 [18]. In our study, the proportion of respondents who maintained a healthy body weight was 56.8%, which is lower than the rate of non-obesity (i.e., body mass index <25 kg/m^2^, 62% in 2020) as reported by the KNHANES [18]. Compliance with these 2 health behaviors—namely, engaging in physical activities and maintaining a healthy body weight—has declined during the COVID-19 pandemic. In our study, one-third and onequarter of study participants answered that they exercised less and gained weight, respectively, due to COVID-19. Lack of exercise, eating out, late-night meals, fast-food consumption, and extended sedentary hours were associated with weight gain among adults [25] and children and adolescents [26]. According to the Korea Community Health Survey (KCHS), the proportion of people who engaged in moderately vigorous physical activity decreased from 24.7% in 2019 to 19.7% in 2021.

Some infection-related cancers, including cancers of the liver and cervix, particularly those associated with HBV and HPV, are preventable through vaccination, health education for safe gender, and screening. The HBV vaccination was introduced into the NIP in Korea in 1995 [27]. The HBV vaccination rate for infants in recent years was up to 98% [28]. Thus, the seroprevalence of hepatitis B surface antigen declined from 4.6% in 1998 to 2.4% in 2020 for those aged 10 years or older. Furthermore, it is much lower among adolescents (0.2%) [18,29]. In our study, a substantial proportion of participants (84%) knew that HBV was a risk factor for liver cancer, and half (51%) of the participants were immunized for HBV. The reasons for not vaccinating were “do not think that vaccination is necessary” (42%) and “do not know the eligibility for vaccination” (34%) (data not shown).

HPV vaccines have been used in Korea since 2007. Since June 2016, HPV vaccination has been included in the NIP [27]. The awareness of HPV vaccination has improved; it was very low (6%) in 2007, the year of the first introduction of HPV vaccines into the Korean market [30], but increased to 37% in 2016, when the HPV vaccine was introduced into the NIP [31], and 50% in 2021. However, as the awareness and practice of HPV vaccination were still the lowest among the 10 recommendations, HPV vaccination has excellent potential for policy interventions to raise awareness. In 2022, the population eligible for HPV vaccination through the NIP was expanded from 12-year-old girls to those aged between 12 years and 17 years and low-income women aged between 18 years and 26 years [32]. Safe gender (i.e., having no gender, minimizing the number of sexual partners, and using condoms) is recommended to reduce the risk of exposure to HBV and HPV, which are sexually transmittable. Although the awareness of safe gender was relatively low compared to that of other recommendations (63%), the proportion of practice was high (86%), since no sexual activity was included in safe gender.

In Korea, both opportunistic and organized cancer screening is available. The National Cancer Screening Program provides fully or partially funded screening for stomach, liver, colorectal, breast, cervix, and lung cancers [33]. In our study, the levels of awareness (96%) and practice (65%) of cancer screening were higher than those of other recommendations and were minimally affected by the COVID-19 pandemic. However, the practice of cancer screening has stagnated in recent years.

In this study, we found differences in awareness and practice among regions; for example, the level of practice was higher in Daegu, Gyeonggi, and Gyeongnam, while it was lower in Ulsan. Regional differences in health-related behaviors have also been found in other surveys, such as the KCHS [34]. The underlying reasons for such differences require further investigation.

Our study had several limitations. First, information was collected based on self-reporting, which can have recall bias, especially regarding vaccination history. Second, awareness and practice could be different in non-respondents. The response rate for the 2021 survey was 17.1%. Low participation rates could lead to non-response bias. Third, even though we designed the proportional quota sampling according to geographical areas, age, and gender for representativeness, the sample size could have been insufficient, especially for the previous surveys from 2007 to 2016 with 1,000-2,000 participants in each survey.

Despite these limitations, our study had strengths. It was based on nationwide, regularly conducted, population-based face-to-face interview surveys. The levels of awareness and practice estimated from this study represent the general population and can be used to monitor the effects of cancer prevention programs.

In conclusion, we found that the awareness levels regarding cancer prevention were high, while the practice levels were low. The recommendation regarding not smoking and avoiding secondhand smoke showed higher awareness and practice levels than other recommendations. Neither the awareness and practice levels of cancer prevention nor the recommendations have changed significantly in the last 15 years. However, the awareness of HPV vaccination has increased, while the awareness of diet-related recommendations has decreased. The various policy implementations, campaigns, and social events in previous years might not have raised public awareness sufficiently to encourage people to practice these recommendations. As we have learned from tobacco control, which achieved decreased smoking rates and changed norms, enhanced policies and programs are needed to promote physical activity, a healthy body weight, a balanced diet, and avoidance of alcohol consumption to prevent cancer.

## Figures and Tables

**Table 1.** The awareness and practice of cancer prevention according to the general characteristics of study participants in the Cancer Prevention Awareness and Practice Survey in 2021

Characteristics	n (%)	Awareness, %	Practice, %
All	4,000	80.3	45.0
Gender			
Men	2,024 (50.6)	81.3	43.6
Women	1,976 (49.4)	79.3	46.4
Age (yr)			
20-29	682 (17.1)	73.9	23.8
30-39	683 (17.1)	79.5	34.0
40-49	832 (20.8)	82.9	44.4
50-59	873 (21.8)	81.8	53.8
60-74	930 (23.3)	81.8	60.9
Region			
Metropolitan cities	2,105 (52.6)	82.3	45.0
Small and medium cities	1,591 (39.8)	78.9	46.3
Counties	304 (7.6)	74.0	37.5
Marital status			
Married	2,724 (68.1)	82.8	52.6
Single	1,173 (29.3)	75.4	26.4
Widowed/separated/divorced	103 (2.6)	69.9	54.4
Education level			
Middle school or less	162 (4.0)	79.0	64.8
High school	1,490 (37.3)	81.0	50.5
College or more	2,348 (58.7)	79.9	40.1
Monthly income (million Korean won)			
<3	781 (19.5)	75.9	44.0
3-6	2,320 (58.0)	81.9	46.2
≥6	899 (22.5)	79.9	42.7

**Table 2.** The awareness, practice, and PAPM stage of the national cancer prevention guidelines

Cancer prevention recommendations	Awareness	Practice	PAPM stage					
			Preadoption			Refusal	Adoption	
			Unaware	Unengaged	Undecided	Decided not to act	Decided to act	Acting
Do not smoke, and avoid secondhand smoke	95.5	75.6	4.5	6.6	6.7	2.8	6.6	72.8
Eat plenty of fruits and vegetables	92.8	54.7	7.2	10.4	4.9	3.7	21.4	52.5
Eat balanced meals with a colorful diet	93.9	40.8	6.2	13.2	8.2	5.7	27.1	39.7
Do not eat salty food	89.6	66.2	10.5	7.1	8.8	4.2	7.5	62.0
Avoid burnt or charred foods	95.8	87.6	4.3	3.1	2.4	1.2	3.1	86.0
Avoid alcohol consumption	86.4	47.7	13.6	16.9	8.7	7.9	10.7	42.2
Walk or exercise at least 30 min/day at least 5 day/wk	88.7	32.5	11.3	10.8	9.4	5.4	32.0	31.1
Maintain a healthy body weight	87.9	56.8	12.1	5.0	3.7	2.8	24.9	51.6
Receive vaccination against HBV	84.0	51.0	16.0	14.3	7.6	2.6	12.2	47.4
Receive vaccination against HPV	54.0	14.5	46.0	19.0	8.7	5.6	7.0	13.7
Undergo cancer screenings	95.6	65.3	4.4	8.8	4.8	2.4	15.4	64.4
Practice safe gender to avoid contracting STIs	62.8	85.9	37.2	1.9	1.3	0.4	2.3	57.1

PAPM, Precaution Adoption Process Model; HBV, hepatitis B virus; HPV, human papillomavirus; STI, sexually transmitted infection.

## SUPPLEMENTARY MATERIALS

Supplementary Material 1. National Cancer Prevention Guidelines

Supplementary Material 2 The awareness and practice of cancer prevention by region in 2021. SU, Seoul; BS, Busan; DG, Daegu; IC, Incheon; GJ, Gwangju; DJ, Daejeon; US, Ulsan; SJ, Sejong; GG, Gyeonggi; GW, Gangwon; CB, Chungbuk; CN, Chungnam; JB, Jeonbuk; JN, Jeonnam; GB, Gyeongbuk; GN, Gyeongnam; JJ, Jeju.

Supplementary Material 3 Odds ratios and 95% of confidence intervals of the awareness and practice of cancer prevention by region in 2021. The odds ratios were adjusted for age and sex using a logistic regression model.

Supplementary Material 4 The awareness and practice of cancer prevention, 2007-2021 n = number of survey participants The practice was calculated among participants who are aware of cancer prevention in 2007-2009. From 2010, it was calculated among all participants. Trends in the ASRs summarized as APC using a linear model without log-transformation. The APC was calculated using age-standardized rate (ASRs) based on 2015 mid-year population statistics. ^*^ Significantly different from zero (p-value < 0.05)
