# Supplementary material for: Awareness of and practice toward cancer prevention recommendations: results of the Korean National Cancer Prevention Awareness and Practice Survey in 2021
Source: Epidemiol Health. 2022 Aug 26;44:e2022068. doi: 10.4178/epih.e2022068 (PMC9943633; doi:10.4178/epih.e2022068)
Supplement: Supplementary Material 1. — National Cancer Prevention Guidelines [file epih-44-e2022068-Supplementary-1.docx]

**Supplementary Material 1.** National Cancer Prevention Guidelines

1. Don't smoke, and avoid secondhand smoke
2. Eat plenty of fruits and vegetables and eat a balanced meal with a colorful diet
3. Do not eat salty food or burnt food
4. Avoid drinking even as little as one or two glasses of alcohol a day
5. Walk or exercise to the point of sweating at least 30 minutes a day at least 5 days a week
6. Maintain a healthy weight for your body
7. Receive hepatitis B and cervical cancer vaccines according to the vaccination guidelines
8. Practice safe sex to avoid contracting sexually transmitted infectious diseases
9. Follow safety and health rules at work to avoid exposure to carcinogenic substances
10. Get appropriate cancer screenings according to screening guidelines to detect cancer at early stages


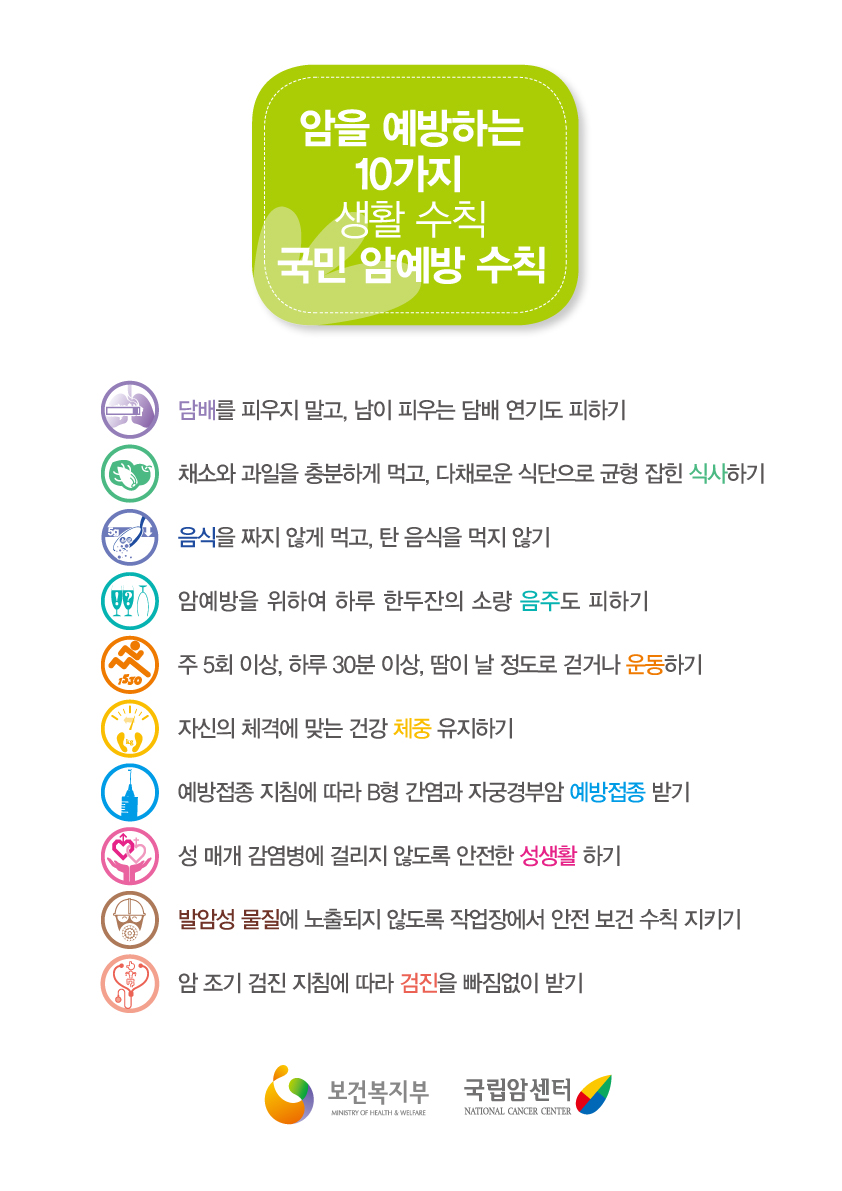


Source: [www.cancer.go.kr](http://www.cancer.go.kr)
