# Supplementary material for: Awareness of and practice toward cancer prevention recommendations: results of the Korean National Cancer Prevention Awareness and Practice Survey in 2021
Source: Epidemiol Health. 2022 Aug 26;44:e2022068. doi: 10.4178/epih.e2022068 (PMC9943633; doi:10.4178/epih.e2022068)
Supplement: Supplementary Material 2 — The awareness and practice of cancer prevention by region in 2021. SU, Seoul; BS, Busan; DG, Daegu; IC, Incheon; GJ, Gwangju; DJ, Daejeon; US, Ulsan; SJ, Sejong; GG, Gyeonggi; GW, Gangwon; CB, Chungbuk; CN, Chungnam; JB, Jeonbuk; JN, Jeonnam; GB, Gyeongbuk; GN, Gyeongnam; JJ, Jeju. [file epih-44-e2022068-Supplementary-2.docx]

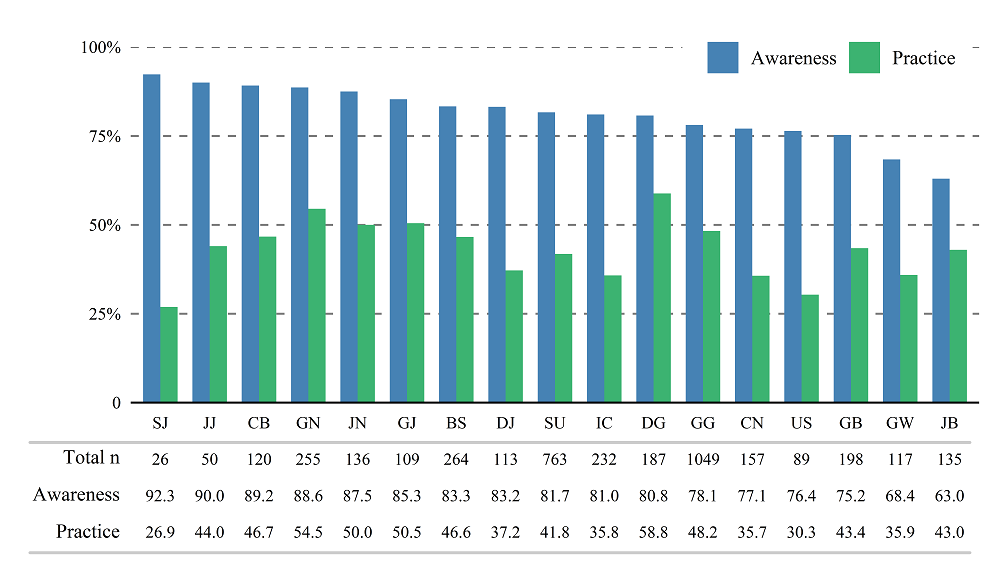


**Supplementary Material 2.** The awareness and practice of cancer prevention by region in 2021.

SU, Seoul; BS, Busan; DG, Daegu; IC, Incheon; GJ, Gwangju; DJ, Daejeon; US, Ulsan; SJ, Sejong; GG, Gyeonggi; GW, Gangwon; CB, Chungbuk; CN, Chungnam; JB, Jeonbuk; JN, Jeonnam; GB, Gyeongbuk; GN, Gyeongnam; JJ, Jeju.
