# Supplementary material for: Awareness of and practice toward cancer prevention recommendations: results of the Korean National Cancer Prevention Awareness and Practice Survey in 2021
Source: Epidemiol Health. 2022 Aug 26;44:e2022068. doi: 10.4178/epih.e2022068 (PMC9943633; doi:10.4178/epih.e2022068)
Supplement: Supplementary Material 3 — Odds ratios and 95% of confidence intervals of the awareness and practice of cancer prevention by region in 2021. The odds ratios were adjusted for age and sex using a logistic regression model. [file epih-44-e2022068-Supplementary-3.docx]

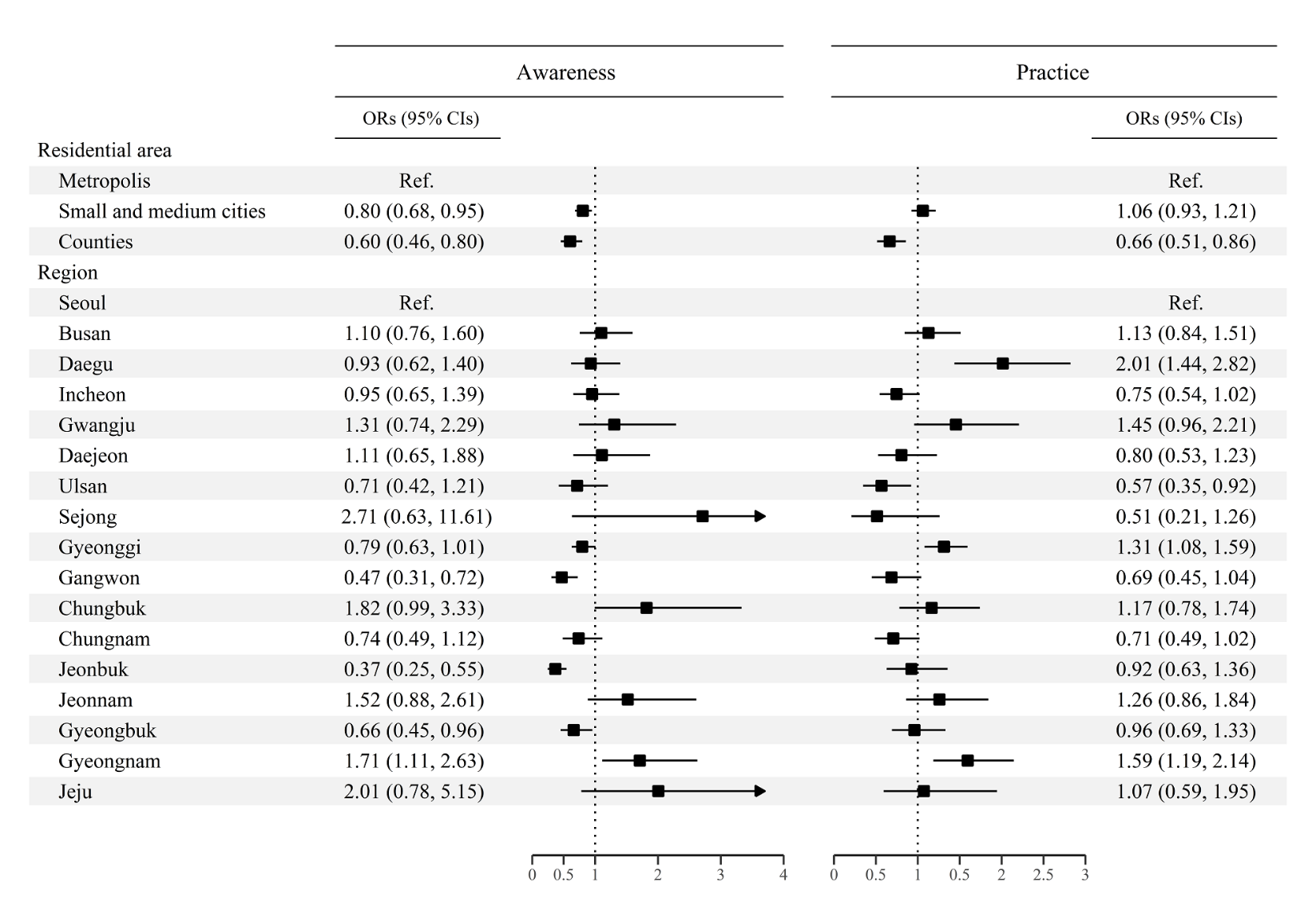


**Supplementary Material 3.** Odds ratios and 95% of confidence intervals of the awareness and practice of cancer prevention by region in 2021. The odds ratios were adjusted for age and sex using a logistic regression model
