# Supplementary material for: Awareness of and practice toward cancer prevention recommendations: results of the Korean National Cancer Prevention Awareness and Practice Survey in 2021
Source: Epidemiol Health. 2022 Aug 26;44:e2022068. doi: 10.4178/epih.e2022068 (PMC9943633; doi:10.4178/epih.e2022068)
Supplement: Supplementary Material 4 — The awareness and practice of cancer prevention, 2007-2021 n = number of survey participants The practice was calculated among participants who are aware of cancer prevention in 2007-2009. From 2010, it was calculated among all participants. Trends in the ASRs summarized as APC using a linear model without log-transformation. The APC was calculated using age-standardized rate (ASRs) based on 2015 mid-year population statistics. * Significantly different from zero (p-value < 0.05) [file epih-44-e2022068-Supplementary-4.docx]

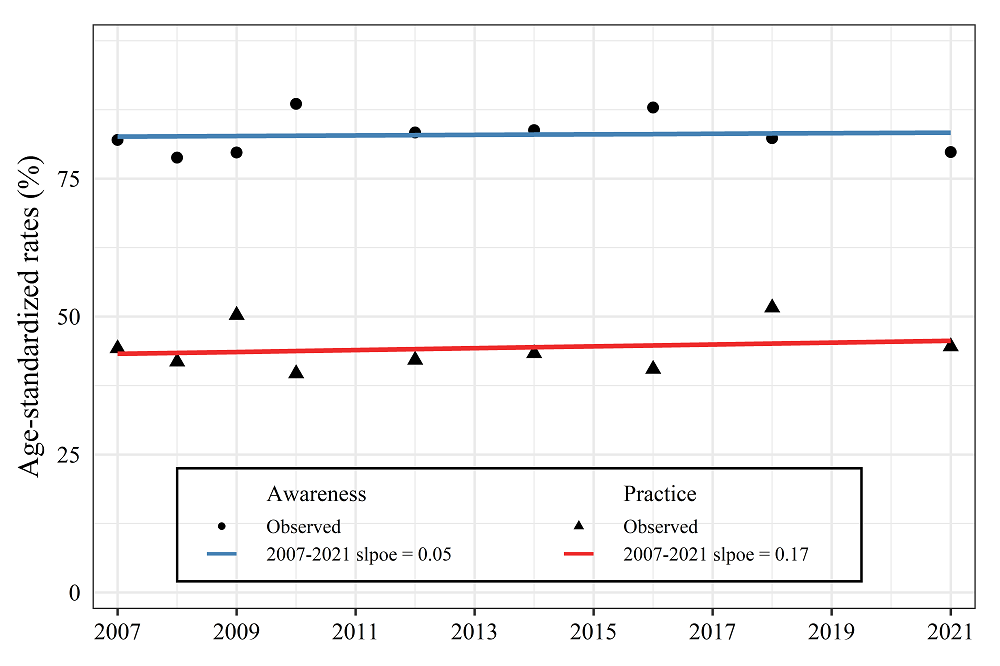


**Supplementary Material 4.** The awareness and practice of cancer prevention, 2007-2021

n = number of survey participants

The practice was calculated among participants who are aware of cancer prevention in 2007-2009. From 2010, it was calculated among all participants. Trends in the ASRs summarized as APC using a linear model without log-transformation. The APC was calculated using age-standardized rate (ASRs) based on 2015 mid-year population statistics.

^*^ Significantly different from zero (p-value < 0.05)
